# MTHFR genetic testing: is there a clinical utility?

**DOI:** 10.1590/1806-9282.20240215

**Published:** 2024-07-19

**Authors:** Aline Cristiane Planello, Darine Villela, Thereza Loureiro

**Affiliations:** 1 Diagnósticos da América S.A., DASA – São Paulo (SP), Brazil.; 2 Faculty of Medicine of Jundiaí, Jundiaí Medical School, Department of Morphology and Pathology – Jundiaí (SP), Brazil.; 3 Universidade Estadual de Campinas, Piracicaba Dental School, Department of Bioscience – Campinas (SP), Brazil.

## OVERVIEW

The *MTHFR* genetic testing investigates two common variants, 677C>T and 1298A>C, and is frequently ordered by several providers due to the association of those variants with several multifactorial diseases, including cancer^1^, autism^2^, mental disorders^3^, cardiovascular diseases^4^, and congenital malformations^5^. The *MTHFR* gene encodes the enzyme methylenetetrahydrofolate reductase (*MTFHR*), which is a crucial component in the one-carbon metabolism pathway that involves folate and homocysteine. Genetic variants in the *MTFHR* may result in decreased enzyme activity, leading to alterations in homocysteine levels. However, it is not clear whether common variants are a major risk factor for diseases because their association with health conditions varies among different populations^5,6^. Notably, some medical companies are currently offering routine *MTHFR* investigation as direct-to-consumer genetic testing. Although this type of testing is becoming increasingly popular due to its accessibility and affordability, it raises concerns about the accuracy of the results and the lack of proper genetic interpretation and counseling for individuals who receive positive results^7^. Several studies have demonstrated the complexity of translating research findings from genetic association studies of common variants in the clinical setting. The relationship between polymorphisms (i.e., common variants) and the risk for multifactorial diseases is complex and influenced by numerous aspects, including ancestry^8^. Furthermore, interpreting polymorphisms, especially in admixed populations, as is the case of the Brazilian population, poses a significant challenge. In this article, we discuss why testing *MTHFR* polymorphisms needs caution, considering that it may not provide significant benefit to patients until more association studies are conducted in diverse populations and the effect of these variants on diseases is fully understood.

## *MTFHR* polymorphisms: challenges in interpretation

Understanding the impact of DNA sequence variants on the *MTHFR* gene and one-carbon metabolism is crucial given the challenge in interpreting genetic variants. Besides environmental factors such as dietary nutrients, the one-carbon metabolism can be affected by either common or rare variants in the *MTHFR*, each presenting different levels of effect^6^. It is expected that most disease-causing variants are rare in the population, with a low allele frequency (AF) of less than 0.1%. The rarer a variant is, the higher the probability of its pathogenicity. The Online Catalog of Human Genes and Genetic (OMIM) database (https://www.omim.org/) describes 10 rare variants in the *MTHFR* gene associated with enzyme deficiency and severe homocysteinemia. In the context of multifactorial diseases, the genetic background that gives rise to an individual's allelic architecture of the disease reflects the contribution of several variants, which individually or in combination contribute to small increments in risk^8,9^. The AF of those small-effect variants is typically higher in the population than the large-effect variants and are often referred to as polymorphisms (AF>1%). Importantly, the effect of a variant will depend on its type, location, and whether the gene is dosage-sensitive or not. Also, the level of effect or penetrance differs among individuals, which can be explained by interactions between different genetic backgrounds and the individual's exposure to environmental factors^8,9^.

**Figure 1:**
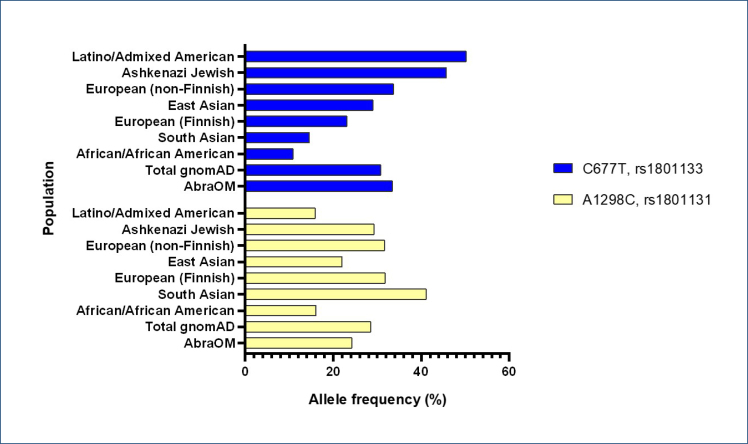
C677T and A1298C allele frequencies from the gnomAD v2 and AbraOM databases. The histogram shows the distribution of the allele frequency of the two most frequent polymorphisms in the methylene tetrahydrofolate reductase gene across different populations.

The C677T and A1298C represent the two polymorphisms in *MTHFR* which are most frequently investigated in clinical practice. Nonetheless, the results of studies showing the association between *MTHFR* polymorphisms and diseases are mixed, and the strength of the association varies depending on the specific polymorphism and population studied. The global AF of the C677T in the Genome Aggregation Database (gnomAD) (https://gnomad.broadinstitute.org/) is 30% and reaches 50% in Latino/Admixed American population, in the Brazilian Genomic Variants database (ABraOM) (https://abraom.ib.usp.br/). However, the AF is 33%. In the case of A1298C, the AF is 28 and 24% in gnomAD and ABraOM, respectively (Figure 1). Both variants present an excess of homozygous carriers in those databases. It is worth mentioning that the *MTHFR* polymorphisms in the homozygous form only reduce enzyme production mildly, which has limited pathogenicity. In addition, studies demonstrate that the individual's ancestry can influence the association of common variants with multifactorial diseases^10-14^. Ancestry-specific variants in combination with environmental factors affect gene–gene and gene–environment interactions, differently from rare variants that lead to Mendelian diseases regardless of the population where they occur. In multifactorial diseases, the variants are pathogenic when combined with other variants and environmental factors, in an additive polygenic model. It is important to acknowledge that most genetic studies have primarily focused on individuals with European ancestry, not capturing the degree of diversity that exists in the global population. As a result, the accuracy of risk estimation of a particular variant for non-European populations can be compromised. Not surprisingly, researchers have demonstrated an association between variants in the *MTHFR* and elevated homocysteine levels only in specific populations. We may then argue that such evidence raises concerns about the clinical utility of *MTHFR* genetic testing.

In particular, the Brazilian population is highly admixed and has one of the most heterogeneous genetic compositions in the world, consisting of three ancestral populations: Native Americans, Europeans, and Africans^15^. A high degree of genetic admixture from these three ancestral populations in Brazil was demonstrated. Indeed, in the ABraOM database, 75% of the studied cohort showed admixture from two or more ancestral populations^16^. Therefore, this level of admixture demands caution when applying findings from polymorphism studies to the Brazilian population. This is especially relevant for *MTHFR*, which could confer susceptibility to various diseases via a polygenic model that can be heterogeneous between ethnic groups. For example, a meta-analysis of 40,173 individuals explored the association between C677T and hypertension risk and showed that the T allele was associated with an increased risk of hypertension in individuals carrying the homozygous TT genotype^17^. However, stratification by ethnicity revealed that the association only existed in Asians and Europeans, but not in Americans and Africans. Another systematic review, which comprised 20 case-control studies, investigated the association between *MTHFR* polymorphisms and the risk of bladder cancer, revealing no overall association, but when data were stratified by ethnicity, both C677T and A1298C were associated with the risk of bladder cancer only in Asians and not Europeans^18^.

Even though meta-analyses can provide a more comprehensive understanding of the association between *MTHFR* polymorphisms and various phenotypes across populations, these studies are not without limitations. The limitations include differences in phenotype definitions and insufficient information on environmental covariates that could affect multifactorial disease models. It is relevant to mention that the evidence and recommendations for *MTHFR* genetic testing have changed over the past years. Currently, the consensus is that, in the absence of elevated homocysteine levels, *MTHFR* variants alone are not a risk factor for any disease. In 2013, the American College of Medical Genetics published a practical guideline advising against routine *MTHFR* genetic testing^19^. Also, many other prominent medical associations discourage the use of the test, including the American College of Obstetrics and Gynecology, the College of American Pathologists, the American Academy of Family Physicians, and the American Heart Association^20^. Despite the lack of evidence for clinical utility, testing for MTHFR polymorphisms remains widespread and providers continue to order this unwarranted test. Moreover, the application of direct-to-consumer genetic testing for *MTHFR* complicates the translation of variant associations, as there is no consideration for the population where those tests are being applied and no health professionals to elucidate the complexity of any variant association. Thus, these limitations raise concerns about the potential harm that may result from individuals making uninformed decisions about their health, being crucial to carefully consider them when utilizing direct-to-consumer genetic testing for clinical purposes.

## CONCLUSIONS AND RECOMMENDATIONS

The *MTHFR* polymorphisms, C677T and A1298C, may or may not be associated with elevated homocysteine levels and the risk for multifactorial diseases. *MTHFR* genetic testing is not likely to provide accurate estimates of disease risk, particularly in highly admixed populations such as the Brazilian population. These variants should be considered part of an additive polygenic model of genes and environment, rather than high penetrant variants because the relationship between *MTHFR* polymorphisms and homocysteine levels or risk for diseases may depend on the presence of other genetic variants in the same individual. Accordingly, genotyping of these two *MTHFR* polymorphisms may not provide significant benefit to patients until further association studies are conducted in diverse populations. If the goal is to correct elevated homocysteine levels through supplementation, it may be more reasonable to test homocysteine levels directly before performing a genetic test for *MTHFR*.
